# A New Strategy Against Peri-Implantitis: Antibacterial Internal Coating

**DOI:** 10.3390/ijms20163897

**Published:** 2019-08-09

**Authors:** Francesco Carinci, Dorina Lauritano, Carlo Alberto Bignozzi, Daniele Pazzi, Valentina Candotto, Paulo Santos de Oliveira, Antonio Scarano

**Affiliations:** 1 Department of Morphology, Surgery and Experimental Medicine, University of Ferrara, 44121 Ferrara, Italy; 2 Department of Medicine and Surgery, Centre of Neuroscience of Milan, University of Milan-Bicocca, 20900 Milan, Italy; 3 Department of Chemical and Pharmaceutical Sciences, University of Ferrara, 44121 Ferrara, Italy; 4 Department of Biomedical, Surgical and Dental Sciences University of Milan, 20122 Milan, Italy; 5 Department of Oral Implantology, Dental Research Division, College Ingà, UNINGÁ, Cachoeiro de Itapemirim 29312, Brazil; 6 Department of Oral Science, Nano and Biotechnology and CeSi-Met University of Chieti-Pescara, 66100 Chieti, Italy

**Keywords:** peri-implantitis, implant dentistry, coating, chlorhexidine gluconate, bacterial loading

## Abstract

The bacterial biofilm formation in the oral cavity and the microbial activity around the implant tissue represent a potential factor on the interface between bone and implant fixture that could induce an inflammatory phenomenon and generate an increased risk for mucositis and peri-implantitis. The aim of the present clinical trial was to investigate the bacterial quality of a new antibacterial coating of the internal chamber of the implant in vivo at six months. The PIXIT implant (Edierre srl, Genova Italy) is prepared by coating the implant with an alcoholic solution containing polysiloxane oligomers and chlorhexidine gluconate at 1%. A total of 15 healthy patients (60 implants) with non-contributory past medical history (nine women and six men, all non-smokers, mean age of 53 years, ranging from 45–61 years) were scheduled to receive bilateral fixed prostheses or crown restorations supported by an implant fixture. No adverse effects and no implant failure were reported at four months. All experimental sites showed a good soft tissue healing at the experimental point times and no local evidence of inflammation was observed. Real-Time Polymerase Chain Reaction (PCR) analysis on coated and uncoated implants showed a decrease of the bacterial count in the internal part of the implant chamber. The mean of total bacteria loading (TBL) detected in each PCR reaction was lower in treated implants (81,038 units/reaction) compared to untreated implants (90,057 units/reaction) (*p* < 0.01). The polymeric chlorhexydine coating of the internal chamber of the implant showed the ability to control the bacterial loading at the level of the peri-implant tissue. Moreover, the investigation demonstrated that the coating is able to influence also the quality of the microbiota, in particular on the species involved in the pathogenesis of peri-implantitis that are involved with a higher risk of long-term failure of the dental implant restoration.

## 1. Introduction

Dental implants are widely used to rehabilitate the edentulous area [1,2,3]. Implant rehabilitation is completely successful when there is no bone resorption around the implant fixture [4,5,6]. Lee et al. indicated that prosthetic loading or bacterial infection could be related to the implant failure and they reported that the prevalence of mucositis is 29.48% and that of peri-implantitis is 9.25% [7].

Osseointegration can be affected by oral conditions, in particular, the micro-gap at the implant–abutment connection (IAC) represents a site for dental plaque aggregation favoring bacterial leakage, which can increase inflammatory cells at the level of the IAC, causing peri-implantitis [8,9]. Two-piece implants unavoidably present a micro-gap between the implant and the abutment. These spaces, once early colonized, may constitute a bacterial reservoir that could subsequently contaminate the implant’s surroundings and interfere with the peri-implant tissues’ health. The presence of a micro-gap, and thus a reservoir of bacteria, when in close relation to the bone, may have a role in bone loss [10]. 

The bacteria found at the IAC level can be both anaerobic and facultatively anaerobic, depending on the features of the microhabitat. In addition, patients at risk of periodontal disease have a higher risk of peri-implantitis [11]. Periodontopathogenic bacteria and in particular the species of “red complex” (*porphyromonas gingivalis*, *tanerella forsythia, treponema denticola),* and other bacteria, such as *Corinebacterium rectus* and *Fusobacterium nucleatum*, cause peri-implant tissue inflammation, which may lead to the destruction of the peri-implant bone, resulting in implant loss [12]. The treatment of peri-implantitis has unpredictable results, while the control of bacterial plaque is crucial [12]. Peri-implantitis is usually associated with gram-negative bacteria similar to those that cause periodontal disease [11,12]. Peri-implantitis, such as periodontal disease, is the result of bacterial insult and the subsequent host response. Some studies have shown that bacterial species of periodontal disease are very similar to those that cause peri-implantitis [11,12,13].

In this way, it has been reported that a clinical history of periodontitis is related with a higher risk for peri-implant infections [11,12]. 

Different analytical methods have been used for a microbiological characterization of microbial species and bacterial quality in peri-implantitis [14]. Polymerase chain reaction (PCR) method is reported as rapid and cost-sensitive for a microbial quantitative evaluation of the oral biofilm and it is able to determine the total bacteria loading (TBL) and identify different bacterial species of the cluster related to the peri-implantitis, such as *Corinebacterium rectus* and *Fusobacterium nucleatum* [14,15,16,17,18]. Many different methods have been described to increase the peri-implant tissue healing and minimize the inflammation reaction around the dental implant fixture.

The prosthetic connection between implant and abutment interface could represent a critical point for a potential microbial colonization that is able to feed an infection and an inflammatory reaction at the level of the peri-implant interface [9].

It has been reported that treatment with an antibacterial coating characterized by chlorhexidine gluconate is able to reduce the bacterial loading in vitro [15].

The aim of the present clinical trial was to investigate the antibacterial activity of a new antibacterial coating of the internal chamber of the implant in vivo at six months.

## 2. Materials and Methods

### 2.1. PIXIT Implants

The PIXIT implant (Edierre srl, Genova, Italy) is produced by coating the internal part of the implants with an alcoholic solution containing polysiloxane oligomers and chlorhexidine gluconate at 1%.

The product has the ability to bind the titanium surface via interaction of the OH– functionalities present on the polisyloxane chains and the titanium surface (antimicrobial coating composition for dental implant, PCT/IT2015/000142). The role of the alkyl chains present on the siloxane units is that of trapping, through Van der Waals interactions, chlorhexidine molecules, allowing their subsequent slow release when in contact with the aqueous medium.

The coating of the internal chamber of the implants was produced by filling with the PIXIT solution which was left in contact with the surface for at least 10 min followed by draining and implant heating at 60 °C for 45 min. The coating of abutment, healing cups, and screws was obtained by immersion in PIXIT solution for 10 min followed by centrifugation on a sintered glass filter and subsequent heating at 60 °C for 45 min. This protocol was used for the first time in a clinical study. The coating treatment of the implant junction has been tested in a previous in vitro study by Lauritano et al. that reported no bacterial growth at the level of the internal chamber [15]. Moreover, the author reported a continuous release from PIXIT of chlorhexidine in the medium, producing an active action over time of the coating [15].

### 2.2. Antimicrobical Test

Healing cups (Edierre srl) were tested for bacterial leakage. Ten healing caps signed as tests and numbered with 15, 16, 19, 20, 24, 25, 26, 30, 31, 32 and ten healing caps signed as controls and numbered with 13, 14, 17, 18, 21, 22, 23, 27, 28, 29, were tested. The caps were inserted in Petri dishes and contaminated with 100 μLT of a microbial pool consisting of Gram positive and negative bacteria (*Staphilococcus aureus*, *Pseudomonas aeruginosa*, *Escherichia coli*, *Enterococcus hirae*) and *Candida albicans* in a concentration range of 1–5 × 10^5^ cfu/mL. After a 10 min contact time, 18 mL of LB Agar were inserted, and the plates were incubated at 37 °C for 24 h. Contamination was also performed in duplicate on Petri dishes that did not contain healing caps.

### 2.3. Population Inclusion

The protocol for this study was designed in accordance with the Helsinki Declaration (revised version of Tokyo at 2004) and Good Clinical Practice Guidelines. The Inter Institutional Ethics Committee of Faculdade Ingá, UNINGÁ, PR, BRAZIL, No. 153455/2018; CAEE 04609518.6.0000.5220, approved it.

A total of 15 healthy patients (60 implants) with non-contributory past medical history (9 women and 6 men, all non-smokers, mean age of 53 years, ranging from 45–61 years) were included in this study. All patients’ candidates were scheduled to receive bilateral fixed prosthesis or crown restorations supported by implant fixture. The subjects were randomly allotted for each group test and control. All patients signed a written informed consent form. 

The subjects were treated at the Department of Oral Implantology, Center for Advanced Studies, Dental Research Division, UNINGÁ Cachoeiro de Itapemirim, Brazil. All patients underwent a preliminary examination and they underwent Orthopanoramic radiographs and 3D Cone Beam for the surgical treatment planning.

The inclusion criteria were: Edentulous or partly edentulous patients with a unilateral or bilateral loss of teeth, with severe alveolar atrophy and a residual alveolar ridge height of between 2 and 4 mm. The exclusion criteria were: Severe illness, head and neck radiation therapy, chemotherapy, uncontrolled diabetes, uncontrolled periodontal disease, and smoking. Exclusion criteria also included facial or neck inflammatory skin diseases, carotid sinus hyperesthesia, hyperthyroidism, and patients who unilaterally declined undergoing post-operative treatment.

### 2.4. Surgical Procedure

The subjects received prophylactic antibiotic therapy: Two grams of amoxicillin (or clindamycin 600 mg if allergic to penicillin) 1 h before surgery, and they were instructed to rinse with chlorhexidine 0.2% (Curasept, Saronno Italy) for 1 min. All patients were treated under local anesthesia. The standard implant site preparation procedure, as recommended by the implant manufacturer, was used. Implants were placed 2 mm under the crestal bone level. Ibuprofen 400 mg was prescribed two to four times daily during meals, for as long as required. Patients were instructed to use chlorhexidine 0.2% mouthwash for 1 min twice daily for 2 weeks, and to avoid brushing and trauma on surgical sites. Postoperative antibiotics were prescribed only to patients who received bone augmentation procedures: Amoxicillin 1 g twice daily for 6 days. Patients allergic to penicillin were prescribed clindamycin 300 mg twice daily for 6 days. Within 1 week all patients were recalled and checked. Implants were exposed and healing screws placed in the implant after 4 months. Within a further 10 days all patients were recalled, healing screws removed, and a sample of the implant internal chamber microbiota was obtained from a single site by a paper probe. DNA was extracted and purified using standard protocols that include two consecutive incubation with lysozyme and proteinase K, followed by spin-column purification. Healing screws were repositioned at 20 Ncm, and recommended manufacturer values were used.

### 2.5. Real-Time Polymerase Chain Reaction

Primers and probes oligonucleotides were designed based on 16S rRNA gene sequences of the Human Oral Microbiome Database (HOMD 16S rRNA RefSeq Version 10.1) counting 845 entries (Biomers.net GmbH, Ulm, Germany). All the sequences were aligned in order to find either a consensus sequence or less conservate spots. Three real-time PCR runs were performed for each sample.

The first reaction quantified the total amount of bacteria using two degenerate primers and a single probe matching a highly conservated sequence of the 16S ribosomal RNA gene. The second reaction detected and quantified the three red complex bacteria, i.e., *Porphyromonas gingivalis, Tannerella forsythia*, *Treponema denticola*, in a multiplex PCR. The third reaction detected and quantified two members of the orange complex *Fusobacterium nucleatum* and *Campylobacter rectus* and a member of purple complex *Aggregatibacter actinomycetemcomitans*. These reactions included a total of six primers and three probes that were highly specific for each species. Table 1 shows probe and primer sequences used for the amplification. Oligonucleotide concentrations and PCR conditions were optimized to ensure sensitivity, specificity, and no inhibitions in case of unbalanced target amounts. Absolute quantification assays were performed using the Applied Biosystems 7500 Sequence Detection System. The amplification profile was initiated by a 10 min incubation period at 95 °C to activate polymerase, followed by a two-step amplification of 15 s at 95 °C and 60 s at 57 °C for 40 cycles. All these experiments were performed including non-template controls to exclude reagents contamination.

Plasmids containing synthetic DNA target sequences (Eurofin MWG Operon, Ebersberg Germany) were used as standard for the quantitative analysis.

Standard curves for each target were constructed in a triplex reaction, by using a mix of the same amount of plasmids, in serial dilutions ranging from 10^1^ to 10^7^ copies. There was a linear relationship between the threshold cycle values plotted against the log of the copy number over the entire range of dilutions (data not shown). The copy numbers for individual plasmid preparations were estimated using the Thermo NanoDrop spectrophotometer.

The absolute quantification of total bacterial genome copies in samples allowed for the calculation of a relative amount of red complex species. To prevent samples and PCR contamination, plasmid purification and handling was performed in a separate laboratory with dedicated pipettes.

### 2.6. Statistical Analysis

The non–parametric Wilcoxon signed rank test was used because data were not normally distributed.

## 3. Result

### 3.1. Clinical Results

A total of 60 implants were positioned, 30 test (implants treated with PIXIT) and 30 control (untreated implants). No adverse effect and no implant failure were reported at four months. All experimental sites showed a good soft tissue healing and no local evidence of inflammation was reported. PCR analysis was performed only in 52 implants (26 treated and 26 untreated implants). PCR analysis on coated and uncoated implants showed a decrease of the bacterial count in the internal part of the implant chamber. The total bacterial load was evaluated by absolute quantification of a conserved ribosomal 16S gene sequences, using a degenerated primers-probe set. The mean of total bacteria loading (TBL) detected with PCR reaction was lower in treated implants (81038 units/reaction) compared to untreated implants (90057 units/reaction) (Table 2 and Table 3). A Wilcoxon signed rank test showed that the effect of treatment was significant (Z = −3.19, *p* = 0.001) (Table 4 and Table 5). Similar results were obtained when amounts of a single bacterial specie was investigated: *Corinebacterium rectus* (Z = −2.75, *p* = 0.006) and *Fusobacterium nucleatum* (Z = −3.74, *p* = 0.001). Postoperative antibiotics were prescribed only to patients who received bone augmentation procedures and only during the implant placement. No antibiotics were prescribed during the healing screws placement. 

### 3.2. Antimicrobical Test Results

**Figure 1:**
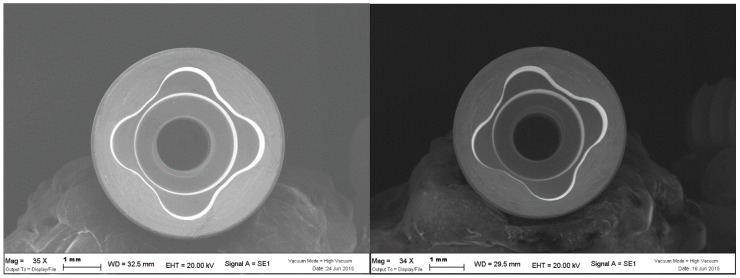
Internal chamber of the fixtures of untreated (**left**) and treated (**right**) with the alcoholic solution containing polysiloxane oligomers and chlorhexidine gluconate at 1% (PIXIT) showing that the coating does not change the internal thickness.

The coating treatment of the implant junction has been tested in a previous in vitro study by Lauritano et al. that reported no bacterial growth at the level of the internal chamber [15]. Moreover the author reported a continuous release from PIXIT of chlorhexidine in the medium, producing an active action over time of the coating [15] (Figure 1).

**Figure 2:**
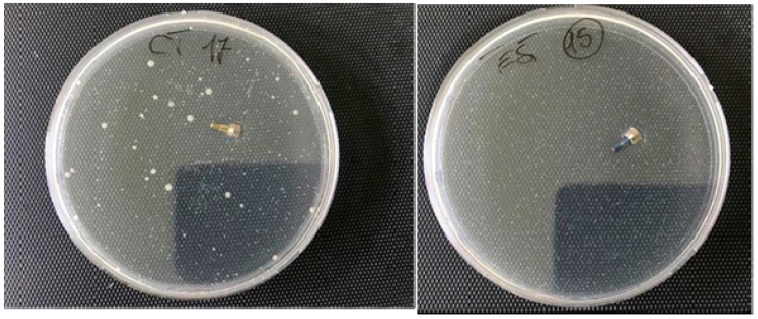
Control (**left**) and test (**right**) healing cups showing the absence of microbial growth in the presence of a contamination of the order of 1–5 × 10^4^ cfu of gram positive, gram negative, and Candida.

Figure 2 shows no bacterial growth after treatment with PIXIT in the test dish. Since the correlation between numbered samples marked as test and controls is not known, an average value of the microbial charge developed in the control plates was evaluated and with respect to this value the reduction of the microbial load which developed in the Petri containing the tests was calculated. In all cases, a considerable reduction of the microbial development in tests compared to samples was observed, in particular the inhibition of the microbial growth was 99% in test sample 15, 95% in test 32, 90% in test 25, 80% in the tests 16, 26, 24 and 31, 60% in test 30, 40% in test 19 and 30% in test 20.

## 4. Discussion

Dental implants are an excellent treatment option for restoring areas that are missing one or more teeth. The majority of dental implants have two components: The implant placed in the bone during the surgical procedure, and the abutment, screwed into the implant to support the prosthetic rehabilitation. These two-piece implant systems present slots and cavities between the implant and the abutment that can act as a trap for bacteria causing the accumulation of pathogens and the onset of peri-implantitis. During the prosthetic rehabilitation, bacterial dissemination is unavoidable, and when the IAC is located at the level of the bone crest, the formation of biofilms in this area causes bone resorption, observed in the early stages of prosthetic load. Bacterial loading is an important factor in peri-implantitis, occurring during soft tissue manipulation of prosthetic rehabilitation. It is clear that peri-implantitis occurs due to the presence of pathogenic microorganisms colonizing the surrounding implant area and the suppression or eradication of these microbes prevents peri-implantitis. The main cause of peri-implantitis consists in the passage of pathogenic bacteria in the abutment-implant gap. The inner spaces are easily colonized, and bacteria may leak out from these spaces through the IAC into the peri-implant area. Peri-implantitis is usually associated with gram-negative bacteria similar to those that cause periodontal disease. Covering IAC surfaces with coatings, by adding biomimetic bioactive substances to improve its biological characteristics, has also been recently investigated [15]. Modifications of IAC using various modalities aim to improve prevention of bacterial infection and promote faster healing times. These aspects are of paramount importance in modern dentistry, since immediate or early loading has become a predictable treatment protocol. 

The presence of a micro gap at the IAC is well known and varies from 1 to 49 μm according to different implant systems and colonization of IAC may cause peri-implant bone resorption [16,17]. The IAC is usually situated under the soft tissue of the gingiva, sometimes very near to the bone. For this reason, the control of bacterial leakage through the IAC can be a major aspect in preventing the infection of the peri-implant tissues. In patients with previous periodontal diseases, the reduction of the passage of pathogenic bacteria should be considered as important, especially because studies have shown that bacterial species of periodontal disease are very similar to the bacteria that cause peri-implantitis [18]. The clinician should keep in mind that the presence of a pathogenic microflora in an initial phase can be associated with a higher risk of peri-implantitis, as per periodontal diseases. 

The prevention of the microbial infection at the level of the implant junction appears the key point for the healing of the peri-implant tissue and a long term maintaining of the dental implant restoration [19,20,21,22].

The coating treatment of the implant junction has been tested in a previous in vitro study by Lauritano et al. that reported no bacterial growth at the level of the internal chamber [15]. Moreover the author reported a continuous release from PIXIT of chlorhexidine in the medium, producing an active action over time of the coating [15]. The protocol adopted in the present study exclude the effect of antibiotic on bacteria proliferations in both groups. The antibiotics used during the implant surgery did not cause a bias in the results because no preoperative o postoperative antibiotics were prescribed during the healing screws placement. In fact, the use of a mucoperiosteal flap in implant surgery does not represent a significant risk for developing bacteremia [23]. Antibiotic prophylaxis in surgery is only indicated when surgery is performed in infected sites, when large foreign materials are implanted, in patients who are at risk of infectious endocarditis, and immuno-compromised patients [24,25]. 

In conclusion, the polymeric coating trapping chlorhexidine in the internal chamber of the implant showed the ability to control the bacterial loading at the level of the peri-implant tissue. Moreover, the investigation demonstrated that the coating is able to influence also the quality of the microbiota, in particular on the species involved in the pathogenesis of peri-implantitis that are involved with a higher risk of long-term failure of the dental implant restoration.

## Figures and Tables

**Table 1:** Probe and primer sequences use for periodontal bacteria amplification.

Periodontal Bacteria	Primer Sequence 5′ -> 3′	Probe Sequence
*Aa*	f-ACCTTACCTACTCTTGACATCCGAA r-ATGCAGCACCTGTCTCAAAGC	AAGAACTCAGAGATGGGTTTGTGCCTAGG
*Pg*	f-CGCGTGAAGGAAGACAGTCC r-CGATGCTTATTCTTACGGTACATTCA	TACGGGAATAACGGGCGATACGAGTATTG
*Tf*	f-CAGCGATGGTAGCAATACCTGTC r- TTCGCCGGGTTATCCCTC	TGAGTAACGCGTATGTAACCTGCCCGC
*Td*	f-AGCTACGGCTCCGCTTCAG r-GATACCCATCGTTGCCTTGGT	AGCTAATGGGACGCGGGCCCAT
*Fn*	f-AGGGTGATCGGCCACAAG r-CACAGAATTGCTGGATCAGACTCT	ACACGGCCCTTACTCCTACGGGAGG
*Cr*	f-TGACGCTAATGCGTGAAAGC r-CTCGACTAGCGAAGCAACAACTAG	TACCCTGGTAGTCCACGCCCTAAACGA
TBL	f-TGGAGCATGTGGTTTAATTCGA r-TGCGGGACTTAACCCAACA	CACGAGCTGACGACARCCATGCA

*Aa: Aggregatibacter actinomycetemcomitans; Pg: Porphyromonas gingivalis; Tf: Tannerella forsythia; Td: Treponema denticola; Fn: Fusobacterium nucleatum; Cr: Campylobacter rectus*; TBL: Total bacterial load.

**Table 2:** Bacterial count observed for treated with the alcoholic solution containing polysiloxane oligomers and chlorhexidine gluconate at 1% (PIXIT) and untreated implants.

	N	Min	Max	Mean	SD
Fn-U	26	0	1639	410	393
Cr-U	26	0	2387	914	758
Cr-T	26	0	192	59	66
Fn-T	26	0	228	81	79
TBL-T	26	37,192	149,707	81,038	45,146
TBL-U	26	40,013	191,064	90,057	53,788

Fn: *Fusobacterium nucleatum; Cr: Campylobacter rectus*; TBL: Total bacterial load; T: Treated implants; U: Untreated implants.

**Table 3:** Statistical analysis in order to verify the distribution of samples. a Lilliefors Correction.

	Kolmogorov-Smirnova ^a^			Shapiro-Wilk		
	Stat	df	*p* Value	Stat	df	*p* Value
Fn-T	0.188	26	0.019	0.857	26	0.002
Cr-T	0.314	26	0.000	0.810	26	0.000
Cr-U	0.270	26	0.000	0.843	26	0.001
Fn-U	0.191	26	0.016	0.874	26	0.004
TBL-T	0.330	26	0.000	0.732	26	0.000
TBL-U	0.304	26	0.000	0.772	26	0.000

**Table 4:** Non Parametric Test. Wilcoxon test. Distribution of ranks. a. FN-U < FN-T; b. FN-U > FN-T; c. FN-U = FN-T; d. CR-U < CR-T; e. CR-U > CR-T; f. CR-U = CR-T; g. TBL-U < TBL-T; h. TBL-U > TBL-T; i. TBL-T = TBL-T.

		N	Mean Rank	Sum
	Neg Rank	4 ^a^	3.75	15.00
	Pos Rank	19 ^b^	13.74	261.00
FN-U – FN-T	Equal values	3 ^c^		
	Tot	26		
	Neg Rank	2 ^d^	5.75	11.50
	Pos Rank	13 ^e^	8.35	108.50
CR-U – CR-T	Equal values	11 ^f^		
	Tot	26		
	Neg Rank	7 ^g^	7.14	50.00
	Pos Rank	19 ^h^	15.84	301.00
TBL-U – TBL-T	Equal values	0 ^i^		
	Tot	26		

**Table 5:** Output of Wilcoxon test. a. Wilcoxon test. b. Based on negative ranks.

	FN-U – FN-T	CR-U – CR-T	TBL-U – TBL-T
Z	−3.741 ^b^	−2.755 ^b^	−3.187 ^b^
Sig. Asint. a 2 code	0.000	0.006	0.001
